# Dr. Leon Gay Lewis

**Published:** 1914-12

**Authors:** 


**Dr. Leon Gay Lewis,** Hahnemann of Chicago 1910, died in the Good Samaritan Hospital, Milwaukee, of typhoid, September 29, aged 27. He formerly served on the staff of the Ernest Wende Hospital of Buffalo.

